# A Rapid and Nondestructive Approach for the Classification of Different-Age Citri Reticulatae Pericarpium Using Portable Near Infrared Spectroscopy

**DOI:** 10.3390/s20061586

**Published:** 2020-03-12

**Authors:** Pao Li, Xinxin Zhang, Shangke Li, Guorong Du, Liwen Jiang, Xia Liu, Shenghua Ding, Yang Shan

**Affiliations:** 1 College of Food Science and Technology, Hunan Provincial Key Laboratory of Food Science and Biotechnology, Hunan Agricultural University, Changsha 410128, China; lipao@mail.nankai.edu.cn (P.L.); zxxhunauyjs@yeah.net (X.Z.); lishangkekk@yeah.net (S.L.); hnndjlw@163.com (L.J.); liuxiaspr@aliyun.com (X.L.); 2 Hunan Agricultural Product Processing Institute, Hunan Academy of Agricultural Sciences, Changsha 410128, China; shhding@hotmail.com; 3 Beijing Work Station, Technology Center, Shanghai Tobacco Group Co. Ltd., Beijing 101121, China; nkchem09@mail.nankai.edu.cn

**Keywords:** citri reticulatae pericarpium, portable near infrared spectroscopy, nondestructive analysis, principal component analysis, fisher linear discriminant analysis

## Abstract

Citri Reticulatae Pericarpium (CRP), has been used in China for hundreds of years as a functional food and medicine. However, some short-age CRPs are disguised as long-age CRPs by unscrupulous businessmen in order to obtain higher profits. In this paper, a rapid and nondestructive method for the classification of different-age CRPs was established using portable near infrared spectroscopy (NIRS) in diffuse reflectance mode combination with appropriate chemometric methods. The spectra of outer skin and inner capsule of CRPs at different storage ages were obtained directly without destroying the samples. Principal component analysis (PCA) with single and combined spectral pretreatment methods was used for the classification of different-age CRPs. Furthermore, the data were pretreated with the PCA method, and Fisher linear discriminant analysis (FLD) with optimized pretreatment methods was discussed for improving the accuracy of classification. Data pretreatment methods can be used to eliminate the noise and background interference. The classification accuracy of inner capsule is better than that of outer skin data. Furthermore, the best results with 100% prediction accuracy can be obtained with FLD method, even without pretreatment.

## 1. Introduction

Citri Reticulatae Pericarpium (CRP) has been used in China for hundreds of years as a functional food and medicine. CRP is rich in volatile oil, flavonoids, polysaccharides and alkaloids, which can be used to treat digestive problems and respiratory complaints [1]. The research shows that the longer the storage time is, the higher the medicinal value of CRP is [2], however, the differences between CRPs of different age are not significant. In recent years, unscrupulous businessmen have marketed young-age CRP as old-age CRP, to obtain illegal profits. It is thus urgent to develop a rapid and simple identification technology for different-age CRP samples.

The differences in appearance and smell can be used for identification of different-age CRPs by experienced persons. However, it is difficult for consumers and food inspectors without experience. Numerous studies have reported that some instrumental methods have been developed to identify CRPs of different ages and varieties. For example, gas chromatography-mass spectrometry (GC-MS) and high performance liquid chromatography (HPLC) were applied for the analysis of the volatile oils and bioactive flavonoids in 25 batches of CRP samples of 10 cultivars collected from different regions [3]. A high-performance thin-layer chromatography (HPTLC) method was used to analyze the volatile compound dimethyl anthranilate, while HPLC was used to simultaneously quantify dimethyl anthranilate and three predominant flavonoids in different varieties of CRP samples [4]. Headspace-gas chromatography-ion mobility spectrometry (HS-GC-IMS) was established to discriminate different varieties of CRP samples by their volatile organic compounds (VOCs) [5]. The CRPs within different storage years were analyzed with ultra-high performance liquid chromatography quadrupole/time-of-flight mass spectrometry based metabolomics approach and 31 metabolites, such as aloesone, roseoside, and 7-hydroxy-5,3’,4’-trimethoxyflavone, etc. were identified to distinguish CRPs of different storage years [6]. However, these methods need time-consuming sample preparation, and sometimes it is difficult to detect the subtle differences in different-age CRPs. 

Near infrared spectroscopy (NIRS) is a simple, rapid and non-destructive method, which has been widely used in the analysis of complex samples in the fields of food [7,8,9,10], agriculture [11,12,13] and medicine [14], by detecting the information of hydrogen-containing functional groups such as C-H, N-H, S-H, and O-H ’stretching vibrations. An important development trend in NIRS technology is miniaturization and reduced instrument costs. Many kinds of portable NIRS instruments have been developed for rapid on-site sample analysis [15]. However, due to the low sensitivity of the NIRS instruments and the complexity of the samples, the useful information of the determined components is usually contained in broad spectral peaks. Besides, the spectra are often disturbed by baseline drift and noise. A large number of chemometric methods have been developed to solve these problems. Many spectral preprocessing methods, such as de-bias correction, detrend (DT), standard normal variate (SNV) transformation, maximum and minimum normalization (MinMax), Mean-Center, multiplicative scatter correction (MSC), first-order derivative (1st) and second-order derivative (2nd) and continuous wavelet transform (CWT) are used to eliminate the background and noise interferences in the spectra [16,17,18]. De-bias and DT are two simple preprocessing methods to eliminate the baseline drift [19]. MSC and SNV methods can be used to eliminate the scattering effects of different particle sizes and uneven particle distribution [20,21,22,23]. MinMax and Mean-Center methods can be used to normalize all variables into a certain range [24]. CWT, 1st and 2nd methods are baseline correction methods which subtract the influence of background and baseline drift [25,26]. However, the noise level increases apparently in higher order derivative calculation. Besides, combination preprocessing methods are typically used to remove multiple interferences in the spectra, since a single method can only suppress one certain interference [27]. Variable selection methods can improve the prediction performance, make the calibration reliable and provide simpler interpretation [28,29]. Principal component analysis (PCA) [14,30,31] and Fisher linear discriminant analysis (FLD) [32,33] are used for the establishment of identification model, while partial least-regression (PLS) and related robust techniques [17,30] are used for the quantitative analysis.

Although NIRS technology combined with chemometric methods has been widely applied in the analysis of complex food samples, there are few studies on the identification of different-age CRPs due to the complexity of the sample and no significant difference among the components [6]. The aim of this study is to obtain reliable and accurate identification results of different-age CRPs with portable NIRS instrument and chemometric methods. Spectra of outer skin and inner capsule were obtained directly by the portable NIRS instrument without destroying samples. PCA combined with single and combined pretreatment methods were used for the classification of different-age CRPs. Furthermore, FLD with appropriate data pretreatment methods was discussed for obtaining a satisfactory classification result.

## 2. Materials and Methods

### 2.1. CRP Sample

Different-age CRPs (5, 10, 15, 20 and 25 years) were obtained from Guangdong Fu Dong Hai Co., Ltd. (Zhanjiang, China). The color of outer skin is brown and the color of the inner capsule is light brown. Each CRP is composed of three petals of pericarp (~50 mm diameter) and a petal for each CRP was used directly as the test sample without destroying it. Forty samples were taken from each age group and a total of 200 samples were collected. The samples were individually packed in sealed polyethylene bags and stored under dry conditions. To reduce the effect of sample temperature on the prediction accuracy, the samples were placed at room temperature for 24 h for equilibration.

### 2.2. Instrumentation and Measurements

**Figure 1:**
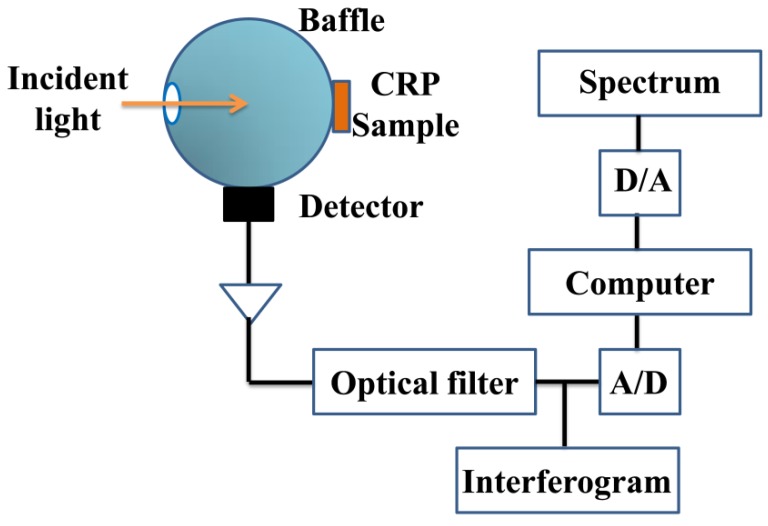
Structure of the near infrared spectroscopy instrument.

The spectra of outer skin and inner capsule were obtained directly using a QuasIR 4000 portable Fourier transform NIRS instrument (Galaxy Scientific, Nashua, NH, USA) in diffuse reflectance mode without destroying the samples. The system consists of a light source, interferometer, fiber optical sensor, InGaAs detector (Galaxy Scientific, Nashua, NH, USA) and data collection card, as shown in Figure 1. The CPR petal was placed directly in the middle of the spot without the container. The selected 200 CPR samples were measured. The measurements were repeated three times and averaged. Each spectrum is composed of 2098 data points recorded from 12,000 to 4000 cm^−1^. 

### 2.3. Data Analysis

The 200 different-age CPR samples were divided into a calibration dataset with 150 samples and a validation dataset with 50 samples by the Kennard-Stone (KS) method. De-bias and DT were used to eliminate the baseline drift in the spectra, while the MSC and SNV methods were used to eliminate the scattering effects. MinMax and Mean-Center methods were applied to normalize all variables into a certain range. CWT, 1st and 2nd methods were used to subtract the influence of background and baseline drift. Combined pretreatment methods, first-order derivative-detrend (1st-DT), first-order derivative-standard normal variate (1st-SNV), first-order derivative-multiplicative scatter correction (1st-MSC), and continuous wavelet transform-standard normal variate (CWT-SNV), continuous wavelet transform-multiplicative scatter correction (CWT-MSC) and standard normal variate- first-order derivative (SNV-1st) were applied in order to further improve the classification accuracy. PCA with single and combined spectral pretreatment methods was used for the classification of different-age CRPs. The spectra were Mean-Centered prior to the creation of the models. To obtain satisfied classification results, FLD method with single and combined pretreatment methods was used. LDA method has the disadvantage that the number of calibration samples must be larger than the number of variables included in the LDA model [34]. Generally, the suggested total number of objects should be equal to at least three to five times the number of variables [35]. In this paper, the PCA method was applied to reduce the multidimensionality and the dataset was transformed into fewer principal components (PCs) before FLD calculation.

The programs were performed using Matlab 2010a (The Mathworks, Natick, MA, USA) and run on a personal computer. The spectral data and results were visualized in Origin 9.0 Software (The OriginLab, Northampton, MA, USA).

## 3. Results and Discussion

### 3.1. Spectra of Different-age CRPs with Single Pretreatment Techniques

**Figure 2:**
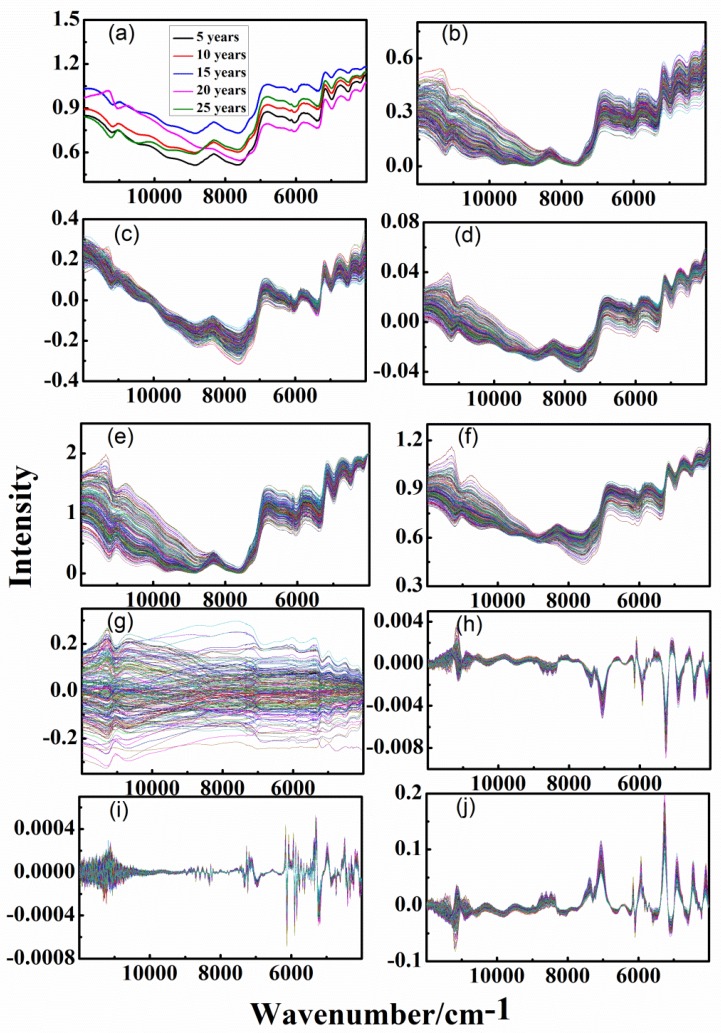
Spectra of outer skin with single pretreatment methods, (**a**–**j**): raw data, detrend (DT), standard normal variate (SNV) transformation, maximum and minimum normalization (MinMax), multiplicative scatter correction (MSC), Mean-Center, first-order derivative (1st) and second-order derivative (2nd) and continuous wavelet transform (CWT).

**Figure 3:**
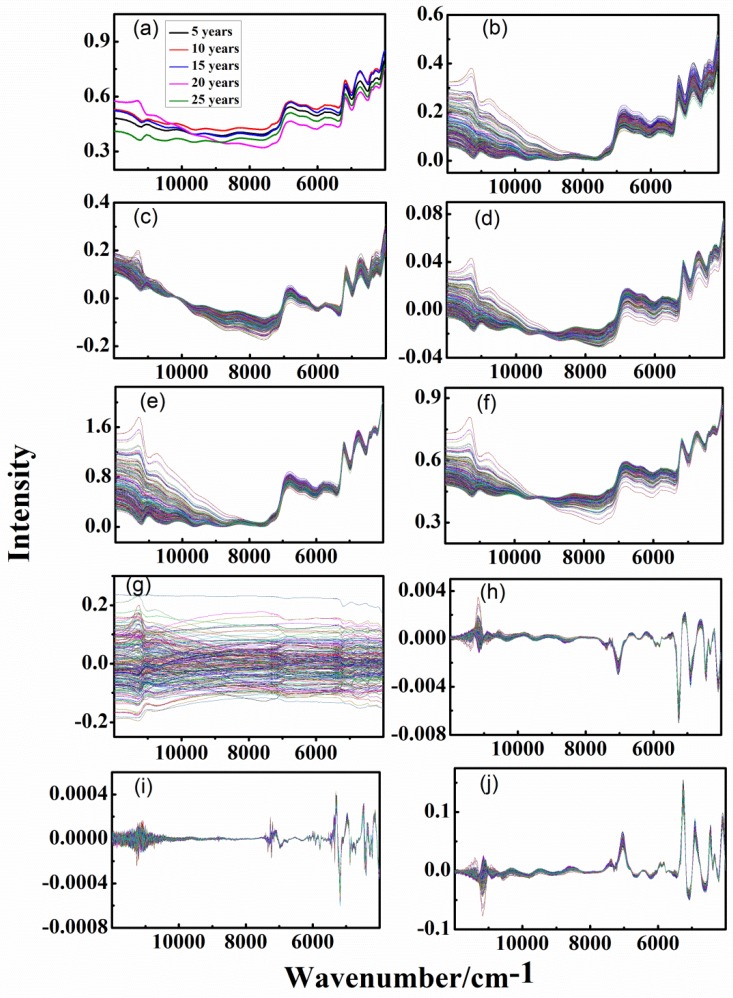
Spectra of inner capsule with single pretreatment methods, (**a**–**j**): raw data, DT, de-bias, SNV transformation, MinMax, MSC, Mean-Center, 1st, 2nd and CWT.

Figure 2a and Figure 3a show the average spectra of each group for the analysis of the outer skin and inner capsule, respectively. It can be seen that there is a very obvious interference of baseline drift in the spectra, due to the rough surface of the CPR samples. There is a slight difference between the spectra trend of outer skin and inner capsule. However, it is difficult to find the difference of different-age CRPs due to the serious interference of overlapping and background. 

Different pretreatment techniques were used to eliminate the background and noise interferences in the spectra. Figure 2b–j and Figure 3b–j show the spectra with DT, de-bias, SNV transformation, MinMax, MSC, Mean-Center, 1st, 2nd and CWT methods, for the analysis of outer skin and inner capsule, respectively. With the help of DT and de-bias methods, the baseline drift interference can be effectively eliminated, shown in Figure 2b,c and Figure 3b,c. The interference of baseline drift is further eliminated with SNV transformation, MinMax and MSC methods, shown in Figure 2d–f and Figure 3d–f. The variant background in the spectra can be removed with 1st, 2nd and CWT methods. Besides, there is very serious noise interference in the wavenumber range of 12,000–10,000 cm^−1^, especially Figure 2i by the 2nd method. This is due to the obvious increase of noise level in higher order derivative calculation. Each spectrum has seven groups of peaks in the wavenumber range of 11,700–10,500, 9000–7600, 7200–6000, 6000–5400, 5300–5000, 5000–4500, and 4500–4150 cm^−1^, which belong to OH second overtone bands, CH second overtone bands, OH first overtone bands, CH first overtone bands, OH combination bands, NH and OH combination bands, and CH combination bands, respectively. In addition, it can be clearly seen that there are differences between the inner and outer spectra in the wavenumber ranges of 9000–7000 and 6400–5600 cm^−1^. However, there is almost no difference among the spectra of different-age CRPs, and the classification of different-age CRPs cannot be achieved with single pretreatment methods.

### 3.2. PCA of Different-Age CRPs with Single Pretreatment Techniques

**Figure 4:**
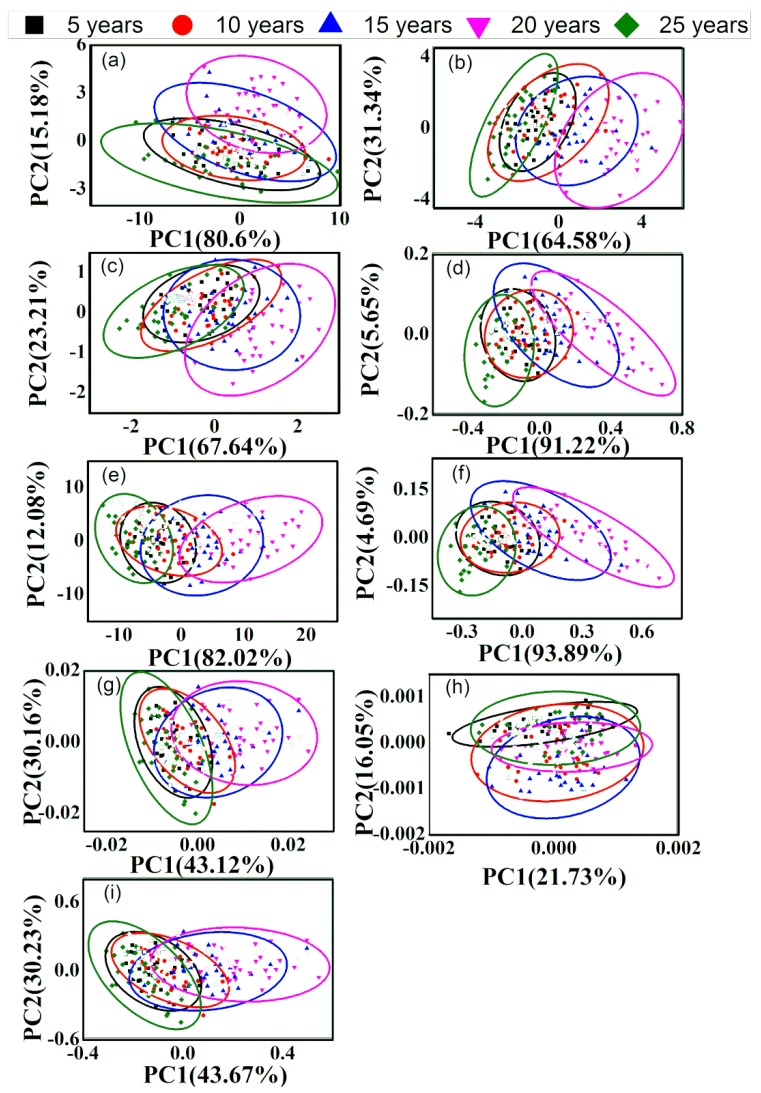
Principal component analysis (PCA) results of outer skin with single pretreatment methods, (**a**–**i**): raw data, DT, de-bias, SNV transformation, MinMax, MSC, 1st, 2nd and CWT.

**Figure 5:**
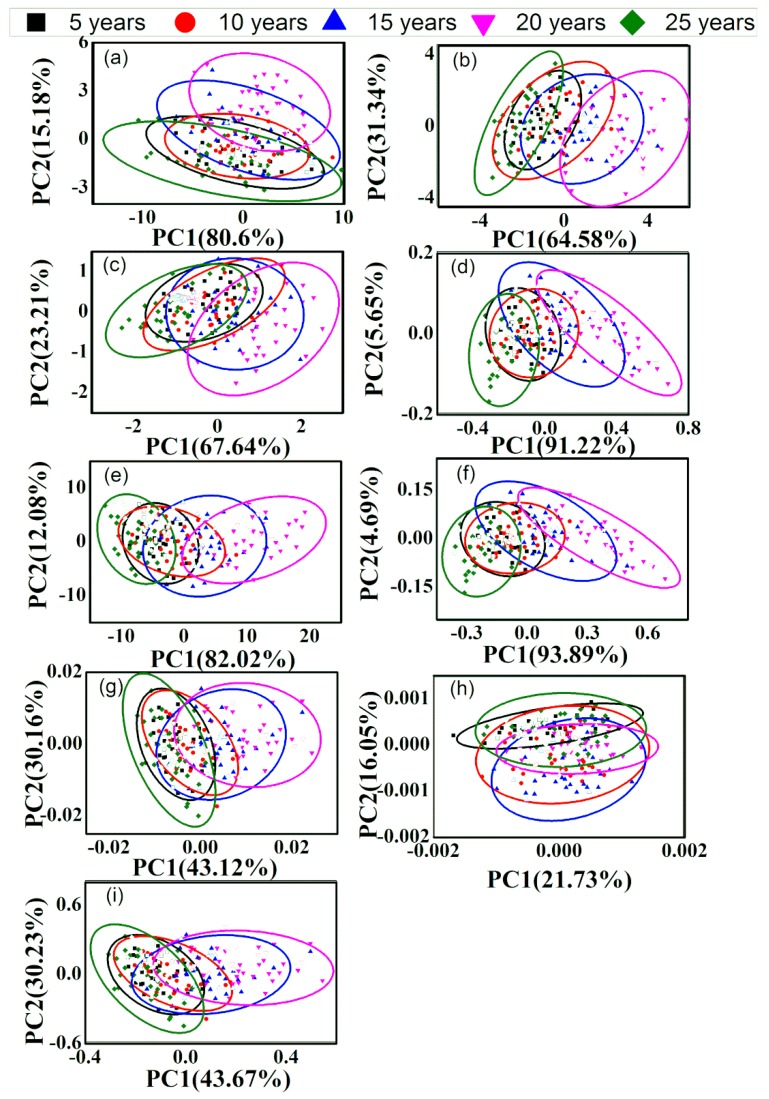
PCA results of inner capsule with single pretreatment methods, (**a**–**i**): raw data, DT, de-bias, SNV transformation, MinMax, MSC, 1st, 2nd and CWT.

In order to discriminate the different-age CRP samples, PCA method was performed. The calibration dataset with 150 samples and the validation dataset with 50 samples were obtained by KS method. Figure 4 and Figure 5 show the classification effect based on the raw spectra and those with single pretreatment methods for the analysis of outer skin and inner capsule data. In the figures, the validation samples are labeled with hollow icons. The first two scores (PC1 and PC2) were used for the classification analysis based on the explanted variances noted in the axis. As shown in Figure 4a and Figure 5a, the five groups are merged together and the classification effect is worse with the raw spectra. The classification accuracies are 2.00% and 6.00%, for the analysis of outer skin and inner capsule data, respectively. Figure 4b–i and Figure 5b–i show the PCA results with DT, de-bias, SNV transformation, MinMax, MSC, 1st, 2nd and CWT methods, for the analysis of outer skin and inner capsule, respectively. The classification results of inner capsule are better than those of outer skin. The best classification accuracy is 10.00% with the SNV transformation and MSC methods for the analysis of outer skin data, while the best classification accuracy is 22.00% with the 2nd method for the analysis of inner capsule data. Therefore, the classification using the spectra with single pretreatment methods may not be feasible.

### 3.3. Spectra of Different-Age CRPs with Combined Pretreatment Techniques

**Figure 6:**
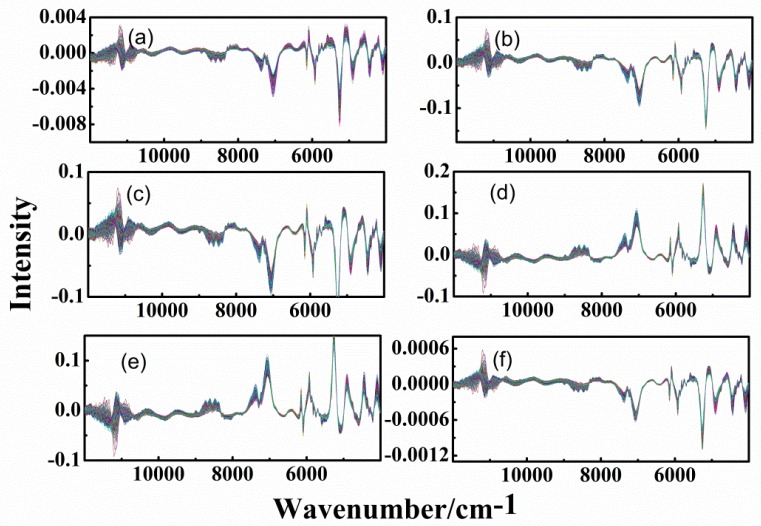
Spectra of outer skin with combined pretreatment methods, (**a**–**f**): first-order derivative-detrend (1st-DT), first-order derivative-standard normal variate (1st-SNV), first-order derivative-multiplicative scatter correction (1st-MSC), and continuous wavelet transform-standard normal variate (CWT-SNV), continuous wavelet transform-multiplicative scatter correction (CWT-MSC) and standard normal variate- first-order derivative (SNV-1st).

**Figure 7:**
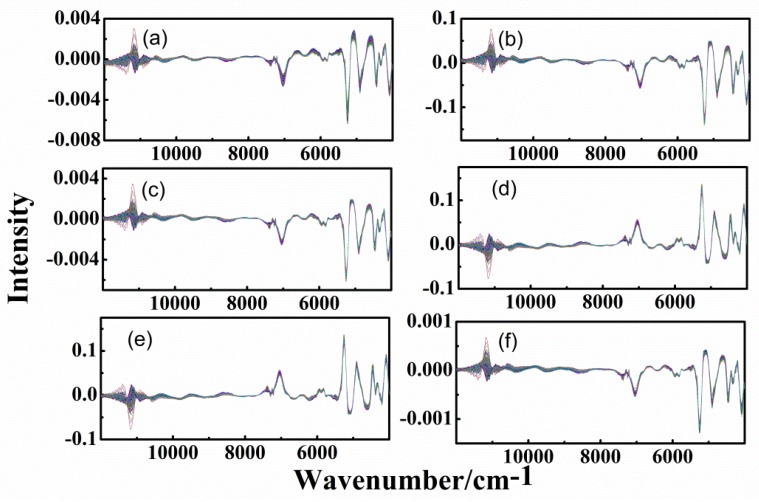
Spectra of inner capsule with combined pretreatment methods, (**a**–**f**): 1st-DT, 1st-SNV, 1st-MSC, CWT-SNV, CWT-MSC, and SNV-1st.

In order to improve the accuracy of classification, combined pretreatment techniques were applied. Figure 6 and Figure 7 show the spectra with 1st-DT, 1st-SNV, 1st-MSC, CWT-SNV, CWT-MSC, and SNV-1st methods, for the analysis of outer skin and inner capsule, respectively. 

CWT and 1st methods can significantly eliminate the background and baseline drift interference in the signal. The changes to signal by CWT and 1st methods are greater than other methods. Therefore, Figure 6a–c,f are similar, while Figure 6d is similar to Figure 6e. Similar results can be obtained for the inner capsule data, shown in Figure 7d–f. In addition, it can be clearly seen that there are differences between the outer skin and inner capsule spectra in the wavenumber ranges of 9000–7000 and 6400–5600 cm^−1^. The noise interference in the outer skin spectra is less than that in the inner capsule spectra.

### 3.4. PCA of Different-Age CRPs with Combined Pretreatment Techniques

**Figure 8:**
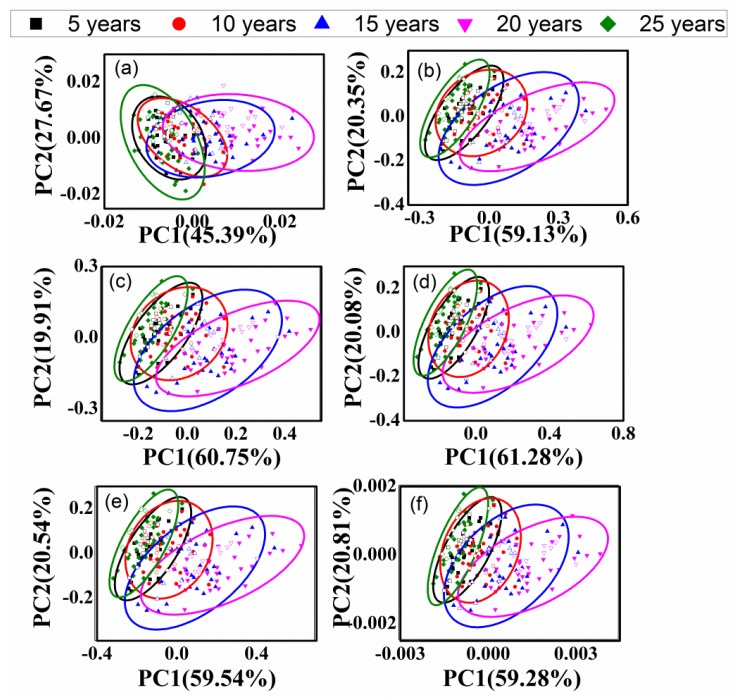
PCA results of outer skin with combined pretreatment methods, (**a**–**f**): 1st-DT, 1st-SNV, 1st-MSC, CWT-SNV, CWT-MSC, and SNV-1st.

**Figure 9:**
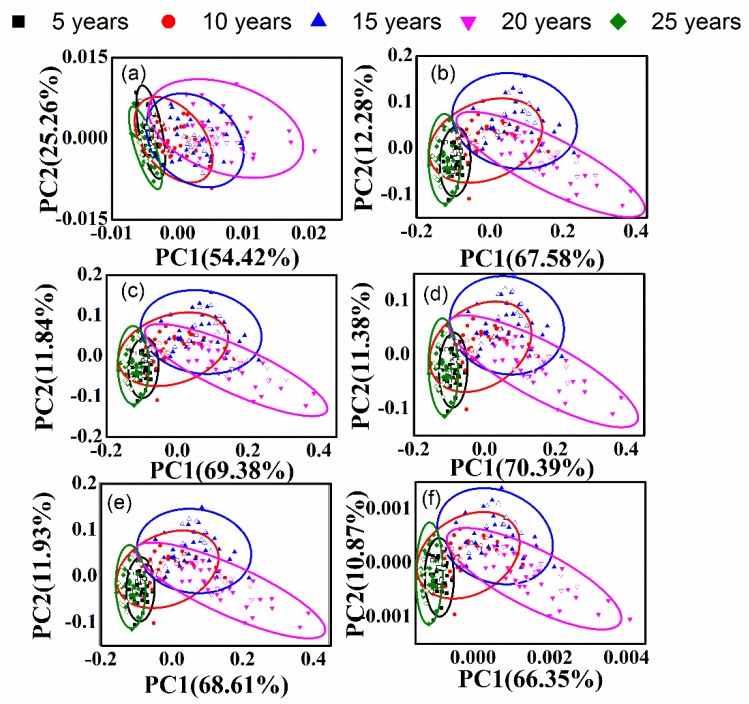
PCA results of inner capsule with combined pretreatment methods, (**a**–**f**): 1st-DT, 1st-SNV, 1st-MSC, CWT-SNV, CWT-MSC, and SNV-1st.

Figure 8 and Figure 9 show the classification effect with combined pretreatment methods for the analysis of outer skin and inner capsule data, respectively. 

The validation samples are labeled with hollow icons, and the first two scores were used for the classification analysis. Figure 8a–c,f are similar, while Figure 8d is similar to Figure 8e. Similar results can be obtained for the inner capsule data, shown in Figure 9a–f. The classification results with combined pretreatment techniques are better than those with single pretreatment techniques, while the classification results of inner capsule are better than those of outer skin. The best classification accuracy is 6.00% with the SNV-1st method for the analysis of outer skin data, while the best classification accuracy is 30.00% with the SNV-1st method for the analysis of inner capsule data. However, the results are still unsatisfactory, even with the combined pretreatment techniques.

### 3.5. FLD of Different-Age CRPs with Pretreatment Techniques

**Figure 10:**
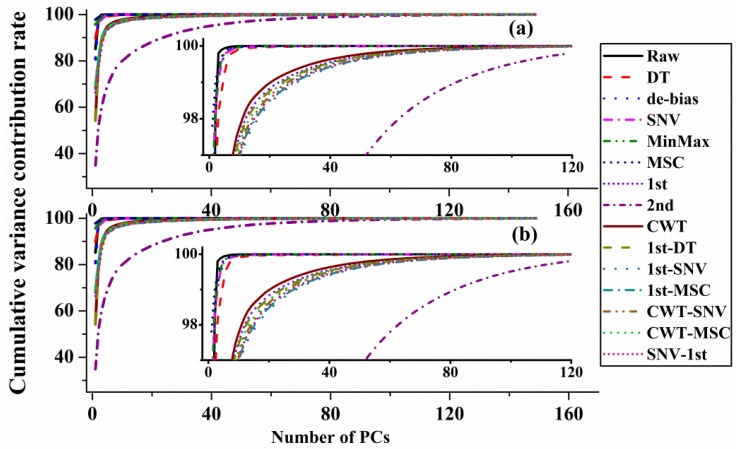
Cumulative variance contribution rates with the increase of principal components number, (**a**): outer skin and (**b**): inner capsule.

As a powerful supervised classification method, the FLD method has been developed to find the optimal boundary between object classes. To make the total number of objects equal to three to five times the number of variables, PCA method was applied to reduce the multidimensionality into fewer PCs before FLD calculation. 200 different-age CPR samples were divided into a calibration dataset with 150 samples and a validation dataset with 50 samples by KS method. Figure 10 is the cumulative variance contribution rates with the increase of PCs number. The value of cumulative variance contribution rate increased rapidly with the increase of PC number and reached a stable high level. For the analysis of spectra with DT, de-bias, SNV transformation, MinMax and MSC methods, most variations (~99%) can be explained when PC number is 5 and variations (~99.99%) can be explained with the PC number 30. For the analysis of spectra with CWT and derivatives methods, variations (~92%) are explained when PC number is 5 and most variations (~99%) can be explained with the PC number 30, except the data with 2nd method (~92%). It is because that the variant background in the spectra is removed with 1st, 2nd and CWT methods. Besides, the noise level increases apparently in higher order derivative calculation. Therefore, 30 PCs were selected for the FLD calculation of both outer skin and inner capsule data.

With the selected PCs, the FLD method was used for the classification analysis of different-age CRP samples, and different pretreatment techniques were applied to optimize the classification model. Table 1 shows the classification accuracies obtained by FLD and different pretreatment methods for the analysis of outer skin and inner capsule spectra. It is clear that the classification accuracies with FLD method are significantly higher than that with PCA method. The identification accuracies of the raw data are more than 96% for the analysis of the outer skin and inner capsule spectra. The result of 2nd method is not satisfactory due to the obvious increase of noise level in higher order derivative calculation. Furthermore, the 100% identification accuracies for the outer skin spectra can be obtained with the raw data or DT, SNV transformation, MinMax and MSC methods, while the 100% identification accuracies for the inner capsule spectra DT, de-bias, SNV transformation, MinMax and MSC methods. 

**Figure 11:**
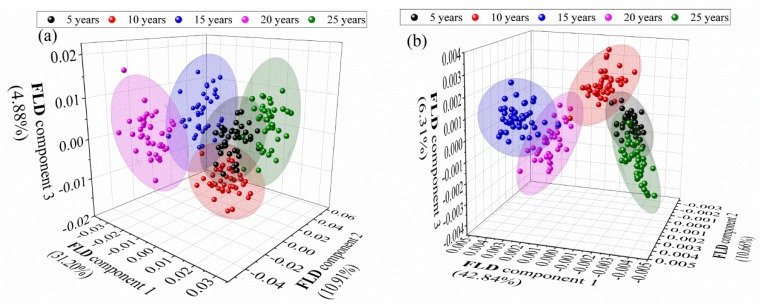
Fisher linear discriminant analysis (FLD) score plots, (**a**): outer skin with FLD method and (**b**): inner capsule with SNV-FLD method.

Furthermore, Figure 11 is the FLD score plots of outer skin spectra with FLD method and inner capsule spectra with SNV-FLD method, and all the five groups were visually separated. Satisfactory results can be obtained with both outer skin and inner capsule spectra, even without any spectral pretreatment. The results demonstrate that, the classification of different-age CRPs can be achieved by the method.

## 4. Conclusions

A rapid and nondestructive method for the classification of different-age CRPs was established using portable NIRS in reflectance mode in combination with appropriate chemometric methods. The spectra of outer skin and inner capsule can be obtained directly without destroying the samples. PCA and FLD methods with single and combined spectral pretreatments were used for the classification of different-age CRPs. Data pretreatment methods can be used to eliminate noise and background interference. The classification accuracy of the inner capsule data is better than that of the outer skin. The best results, with 100% prediction accuracy, can be obtained with FLD method, even without pretreatment. The developed technology can be regarded as a simple, rapid, nondestructive and accurate classification method of different-age CRPs, and has a broad application prospect in the future.

## Figures and Tables

**Table 1:** Classification accuracies obtained by Fisher linear discriminant analysis (FLD) and different pretreatment methods.

Dataset	Pretreatment Method	5 years (%)	10 years (%)	15 years (%)	20 years (%)	25 years (%)	Whole Data (%)
Outer skin data	Raw	100	100	100	100	100	100
	DT	100	100	100	100	100	100
	de-bias	100	100	100	100	90	98
	SNV	100	100	100	100	100	100
	MinMax	100	100	100	100	100	100
	MSC	100	100	100	100	100	100
	1st	100	100	100	100	90	98
	2nd	70	100	80	90	90	86
	CWT	90	100	100	100	100	98
	1st-DT	80	100	100	100	100	96
	1st-SNV	90	100	100	100	90	96
	1st-MSC	100	100	100	100	90	98
	CWT-SNV	90	100	100	100	90	96
	CWT-MSC	90	100	100	100	100	98
	CWT-SNV	100	100	100	100	90	98
Inner capsule data	Raw	90	100	100	100	100	98
	DT	100	100	100	100	100	100
	de-bias	100	100	100	100	100	100
	SNV	100	100	100	100	100	100
	MinMax	100	100	100	100	100	100
	MSC	100	100	100	100	100	100
	1st	80	100	100	100	90	94
	2nd	50	100	90	100	60	80
	CWT	90	100	100	100	90	96
	1st-DT	80	100	100	100	100	96
	1st-SNV	90	100	100	100	90	96
	1st-MSC	90	100	100	100	90	96
	CWT-SNV	90	100	100	100	90	96
	CWT-MSC	90	100	100	100	90	96
	CWT-SNV	90	100	100	100	100	98

## References

[1] Aires A., Carvalho R., Matos M., Carnide V., Silva A.P., Gonçalves B. (2017). Variation of chemical constituents, antioxidant activity, and endogenous plant hormones throughout different ripening stages of highbush blueberry (*Vaccinium corymbosum* L.) cultivars produced in central Portugal. J. Food Biochem..

[2] Shi Q.R., Guo T.T., Yin T.J., Wang Z.Q., Li C.H., Sun X., Guo Y.M., Yuan W.H. (2018). Classification of Pericarpium Citri Reticulatae of different ages by using a voltammetric electronic tongue system. Int. J. Electrochem. Sci..

[3] Luo M.X., Luo H.J., Hu P.J., Yang Y.T., Wu B., Zheng G.D. (2018). Evaluation of chemical components in Citri Reticulatae Pericarpium of different cultivars collected from different regions by GC-MS and HPLC. Food Sci. Nutr..

[4] Li S.Z., Guan X.M., Gao Z., Lan H.C., Yin Q., Chu C., Yang D.P., Liu E.H., Zhou P. (2019). A simple method to discriminate Guangchenpi and Chenpi by high performance thin-layer chromatography and high-performance liquid chromatography based on analysis of dimethyl anthranilate. J. Chromatogr. B.

[5] Lv W.S., Lin T., Ren Z.Y., Jiang Y.Q., Zhang J., Bi F.J., Gu L.H., Hou H.C., He J.N. (2020). Rapid discrimination of Citrus reticulata ‘Chachi’ by headspace-gas chromatography-ion mobility spectrometry fingerprints combined with principal component analysis. Food Res. Int..

[6] Luo Y., Zeng W., Huang K.E., Li D.X., Chen W., Yu X.Q., Ke X.H. (2019). Discrimination of Citrus reticulata Blanco and Citrus reticulata ‘Chachi’ as well as the Citrus reticulata ‘Chachi’ within different storage years using ultra high performance liquid chromatography quadrupole/time-of-flight mass spectrometry based metabolomics approach. J. Pharm. Biomed..

[7] Yu H.Y., Niu X.Y., Lin H.J., Ying Y.B., Li B.B., Pan X.X. (2009). A feasibility study on on-line determination of rice wine composition by Vis-NIR spectroscopy and least-squares support vector machines. Food Chem..

[8] Chen J., Zhu S., Zhao G. (2017). Rapid determination of total protein and wet gluten in commercial wheat flour using siSVR-NIR. Food Chem..

[9] Xiao H., Feng L., Song D., Tu K., Peng J., Pan L.Q. (2019). Grading and sorting of grape berries using visible-near infrared spectroscopy on the basis of multiple inner quality parameters. Sensors.

[10] Xia Z.Y., Sun Y.M., Cai C.Y., He Y., Nie P.C. (2019). Rapid determination of chlorogenic acid, luteoloside and 3,5-o-dicaffeoylquinic acid in chrysanthemum using near-infrared spectroscopy. Sensors.

[11] Tardaguila J., Fernández-Novales J., Gutiérrez S., Paz Diago M. (2017). Non-destructive assessment of grapevine water status in the field using a portable NIR spectrophotometer. J. Sci. Food Agric..

[12] Purcell D.E., O’Shea M.G., Johnson R.A., Kokot S. (2009). Near-infrared spectroscopy for the prediction of disease ratings for fiji leaf gall in sugarcane clones. Appl. Spectrosc..

[13] Jenal A., Bareth G., Bolten A., Kneer C., Weber I., Bongartz J. (2019). Development of a VNIR/SWIR multispectral imaging system for vegetation monitoring with unmanned aerial vehicles. Sensors.

[14] Li P., Du G.R., Cai W.S., Shao X.G. (2012). Rapid and nondestructive analysis of pharmaceutical products using near-infrared diffuse reflectance spectroscopy. J. Pharm. Biomed..

[15] Sandak J., Sandak A., Zitek A., Hintestoisser B., Picchi G. (2020). Development of Low-Cost Portable Spectrometers for Detection of Wood Defects. Sensors.

[16] Rinnan A., Berg F.V.D., Engelsen S.B. (2009). Review of the Most Common pre-Processing Techniques for Near-Infrared Spectra. Trac Trend Anal. Chem..

[17] Bian X.H., Li S.J., Shao X.G., Liu P. (2016). Variable space boosting partial least squares for multivariate calibration of near-infrared spectroscopy. Chemometr. Intell. Lab..

[18] Han X., Huang Z.X., Chen X.D., Li Q.F., Xu K.X., Chen D. (2017). On-line multi-component analysis of gases for mud logging industry using data driven Raman spectroscopy. Fuel.

[19] Liu Y.D., Ying Y.B., Fu X.P. (2005). Study on predicting sugar content and valid acidity of apples by near infrared diffuse reflectance technique. Spectrosc. Spect. Anal..

[20] Geladi P., Macdougall D., Martens H. (1985). Linearization and Scatter-Correction for Near-Infrared Reflectance Spectra of Meat. Appl. Spectrosc..

[21] Barnes R.J., Dhanoa M.S., Lister J.S. (1989). Standard normal variate transformation and de-trending of near-infrared diffuse reflectance spectra. Appl. Spectrosc..

[22] Dhanoa M.S., Lister S.J., Sanderson R., Barnes R.J. (1994). The link between multiplicative scatter correction (MSC) and standard normal variate (SNV) transformations of NIR spectra. J. Near Infrared Spec..

[23] Helland S.I., Naes T., Isaksson T. (1995). Related versions of the multiplicative scatter correction method for preprocessing spectroscopic data. Chemometr. Intell. Lab..

[24] Lu H.Y., Wang S.S., Cai R., Meng Y., Xie X., Zhao W.J. (2012). Rapid discrimination and quantification of alkaloids in Corydalis Tuber by near-infrared spectroscopy. J. Pharm. Biomed..

[25] Savitzky A., Golay M.J.E. (1964). Smoothing and differentiation of data by simplified least squares procedures. Anal. Chem..

[26] Shao X.G., Leung A.K.M., Chau F.T. (2003). Wavelet: A new trend in chemistry. Acc. Chem. Res..

[27] Bian X.H., Wang K.Y., Tan E.R., Diwu P.Y., Zhang F., Guo Y.G. (2020). A selective ensemble preprocessing strategy for near-infrared spectral quantitative analysis of complex samples. Chemometr. Intell. Lab..

[28] Yun Y.H., Li H.D., Deng B.C., Cao D.S. (2019). An overview of variable selection methods in multivariate analysis of near-infrared spectra. Trac Trend Anal. Chem..

[29] Li P., Du G.R., Ma Y.J., Zhou J., Jiang L.W. (2018). A novel multivariate calibration method based on variable adaptive boosting partial least squares algorithm. Chemometr. Intell. Lab..

[30] Wold S., Esbensen K., Geladi P. (1987). Principal component analysis. Chemometr. Intell. Lab..

[31] Biancolillo A., Marini F. (2018). Chapter four-chemometrics applied to plant spectral analysis. Compr. Anal. Chem..

[32] Fisher R.A. (1936). The use of multiple measurements in taxonomic problems. Ann. Eugen..

[33] Yan S., Lai X.X., Du G.R., Xiang Y.H. (2018). Identification of aminoglycoside antibiotics in milk matrix with a colorimetric sensor array and pattern recognition methods. Anal. Chim. Acta.

[34] Brito A.L.B., Brito L.R., Honorato F.A., Pontes M.J.C., Pontes L.F.B.L. (2013). Classification of cereal bars using near infrared spectroscopy and linear discriminant analysis. Food Res. Int..

[35] Wang Y., Mei M.H., Ni Y.N., Kokot S. (2014). Combined NIR/MIR analysis: A novel method for the classification of complex substances such as illicium verum Hook. F. and its adulterants. Spectrochim. Acta A.

